# Rock typing and reservoir characterization of the Messinian Abu Madi Formation in the onshore South Abu El Naga Gas Field, Nile Delta, Egypt

**DOI:** 10.1038/s41598-025-97780-z

**Published:** 2025-05-07

**Authors:** Ahmed W. Al-Shareif, Mohamed A. Khalifa, Bassem S. Nabawy, Mohamed F. Abu-Hashish, Noha M. Hassan

**Affiliations:** 1 Department of Geology, Faculty of Science, Menoufia University, Shibin El Kom, El Menoufia Egypt; 2 El Wastani Petroleum Company (WASCO), 5th Settlement, New Cairo, Egypt; 3 Geophysical Sciences Department, National Research Centre, Cairo, Egypt

**Keywords:** Abu Madi Formation, Petrophysical properties, Hydrocarbon reservoirs, Onshore Nile Delta, Natural gas, Petrol

## Abstract

Exploration of the heterogeneous sandstone reservoirs presents a significant opportunity within the Nile Delta Basin. This study uses the well log, core, and petrographical data to describe the different rock types and characterizes the heterogenous sandstone of the Late Miocene Messinian Abu Madi reservoir as one of the main prolific reservoirs in the South Abu El Naga Gas Field in the Nile Delta. However, accurate assessment of the potential of these complex and heterogeneous sandstone reservoirs requires a meticulous approach. The available data was imported from four wells: SAEN-2, SAEN-4, SAEN-6, and SAEN-9. A total of 35 core plugs, which were derived from two cored intervals in the SAEN-2 well, were used in a well-integrated workflow for reservoir characterization, facies analysis, and rock typing. Core analysis (grain density ‘ρ_g_’, helium porosity ‘∅_He_’, horizontal and vertical permeabilities ‘k_H_ & k_V_’, and water saturation ‘Sw’) and well log data (caliper, gamma-ray, spontaneous potential, PEF, density, neutron, and resistivity logs) provided crucial insights into the lithology, pore systems, and textures. This information allowed us to define the dominant microfacies types as quartz arenite, feldspathic arenite, quartz wacke/wacke, feldspathic wacke, and subfeldspathic wacke. With the core data, it was also possible to estimate the reservoir quality index (RQI), flow zone indicator (FZI), and the effective pore radius (R_35_) from core data, while the net pay thickness, the effective porosity, the shale volume (V_sh_), and the water saturation (Sw) from the well log data. It also enabled the identification of the potential zones of the gas-bearing reservoirs. Hydraulic flow units (HFUs) were established using well logs and core data. These units represent zones with similar fluid flow properties, facilitating the prediction of gas deliverability. Additionally, the flow zone indicator (FZI) that derived from the well logs further characterized the flow regime within the reservoir. Sedimentological studies, including thin section petrography, XRD, and SEM, complemented with the well log interpretation. This integrated workflow provided a comprehensive perspective of the reservoir, including pore structures, mineral composition, and textures. The Abu Madi Formation in the SAEN-9 well, to the northeast of the field, has the lowest net pay (7.3 m), while the SAEN-2 well, in the center of the field, has the highest net pay thickness (16.6 m). The core studies indicate that SAEN samples could be divided into four reservoir rock types (RRTs). The RRT1 has the lowest reservoir quality (0.12 ≤ ∅_He_ ≤ 0.26, 2.4 ≤ k_H_ ≤ 429 mD, 54.9 ≤ Sw ≤ 70.5%, 0.14 ≤ RQI ≤ 1.22 μm, 0.82 ≤ FZI ≤ 3.863 μm, and 1.055 ≤ R_35_ ≤ 11.41 μm), while the RRT4 has the best reservoir quality (0.25 ≤ ∅_He_ ≤ 0.28, 2680 ≤ k_H_ ≤ 4893 mD, 45.4 ≤ Sw ≤ 55.3%, 3.24 ≤ RQI ≤ 4.13 μm, 9.72 ≤ FZI ≤ 10.59 μm, and 34.668 ≤ R_35_ ≤ 44.78 μm). This study demonstrates the effectiveness of an integrated approach in comprehensively assessing the potential gas-bearing reservoirs and defining their quality in the Abu Madi Formation in the Nile Delta, which is characterized by very good reservoir quality (net pay thickness = 7.3–16.6 m, av. porosity = 23.3–30.35%, and av. water saturation = 31.7–64.0% for the various wells). The findings contribute significantly to optimizing exploration and development strategies for gas-bearing hydrocarbon resources in the Nile Delta Basin, especially for the Abu Madi reservoir.

## Introduction

Sandstones are known to constitute approximately 20–25% of sedimentary rocks and account for 50% of the conventional hydrocarbons in global reservoirs^1^. It is challenging to figure out the depositional model and the facies distribution of the deltaic sandstones are due to their heterogeneity and complexity^2^.

The Nile Delta of Egypt remains a critical source for the natural gas, fueling domestic needs and driving exploration for further growth^3^. Recent discoveries like Zohr underscore the region’s exceptional potential, solidifying Egypt’s role as a major gas exporter^4^.

The gas reservoirs in the Nile Delta exhibit a stratigraphic nature, characterized as stratigraphic trap types. They contain a lot of clay minerals, which could affect how accurate petrophysical assessments and calculations of reservoir parameters are. This could lead to inaccurate estimates of hydrocarbon reserves. Characterizing reservoirs is essential in shaly sand formations that exhibit significant lithofacies variability^5^. The relatively high amounts of clay and conductive minerals in these reservoirs make their deep resistivities much lower. This is why they are called low-resistivity pay. Low resistivity pay in reservoirs can be mistaken for high water content because of the low electrical resistivity that comes with it. Low water cut, on the other hand, can lead to hydrocarbon production. According to some research, this might be because of the high level of irreducible water saturation caused by clay minerals, which have very small pores with a lot of surface area^6–8^.

**Fig. 1:**
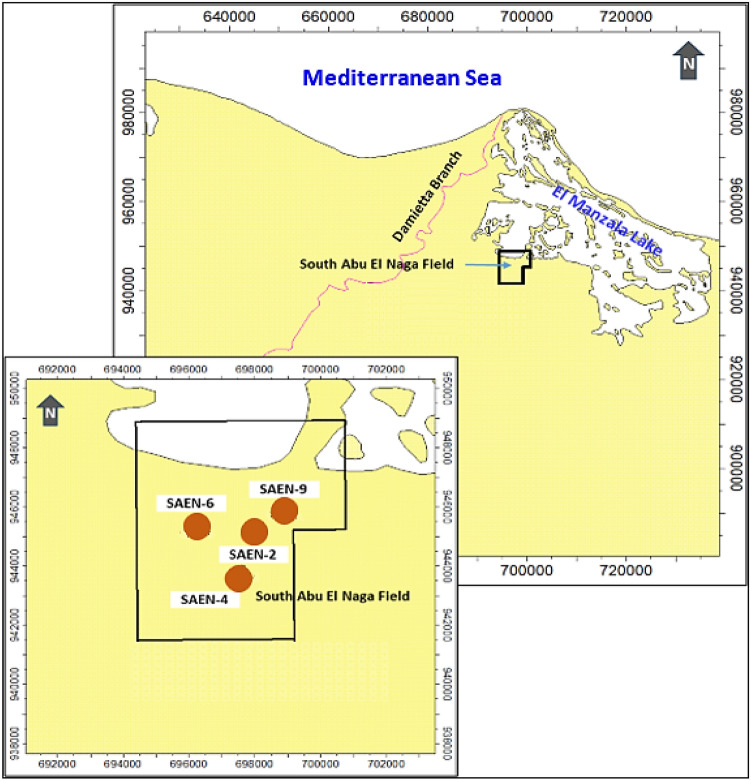
Location map of the onshore South Abu El Naga Gas (SAEN) Field in east of the offshore Nile Delta.

The Nile Delta has a significant geological history and is a vital natural gas resource for Egypt due to extensive sediment deposition. The study area is situated in the eastern Nile Delta Onshore, near El Manzala Lake’s western edge and east of the Damietta Nile branch. It lies between latitudes 31°11′00″ and 31°15′00″, and longitudes 31°50′00″ to 31°5400″, encompassing an area of approximately 8.73 km^2^ (Fig. 1). This study utilizes geological data, well logs, and core samples to characterize Late Miocene Messinian reservoir properties and predict their distribution and quality.

Core tests are the primary method for studying petrophysical properties; however, it is unlikely to obtain plugs from every well to understand the rock properties and stratigraphic accumulation patterns^9,10^. Rock type and pore throat radius dominate the relationship between porosity and permeability. Porosity and permeability can be determined directly from core analysis or NMR^11^. The pore characteristics are one of the quite considerable properties in reservoir evaluation^12^. It influences the storage mechanisms and the properties of reservoir fluids in permeable units. Conversely, pore structure plays a crucial role in determining petrophysical properties and multiphase flow characteristics of reservoir rocks. A new empirical relationship between porosity, and air permeability has been developed to estimate the effective pore throat radius (R_35_) by Winland at 35% mercury saturation for a group of sandstones and carbonates samples^13^.

## Geological setting

**Fig. 2:**
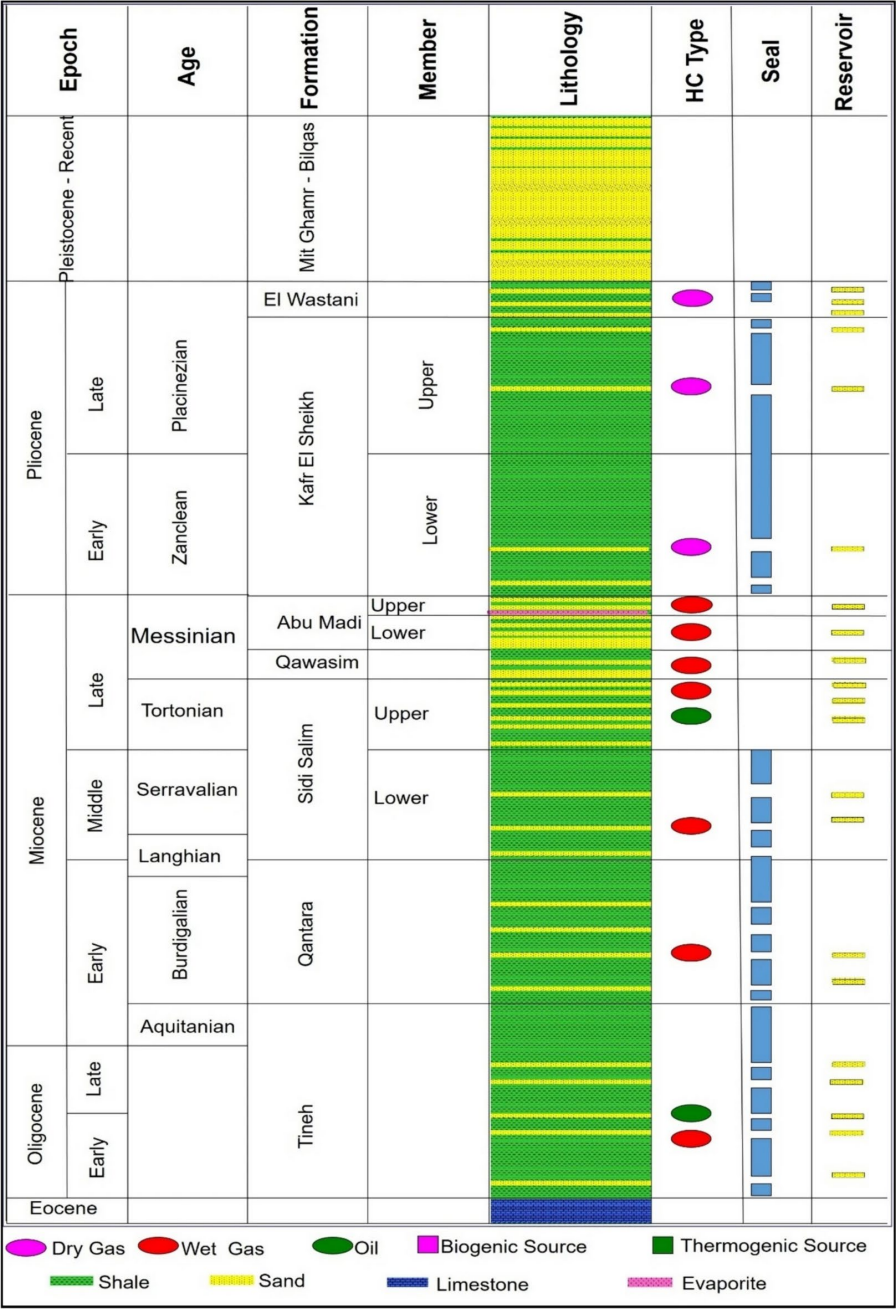
General view of the lithostratigraphic sequence (Eocene-Holocene) and situation of the oil and gas reservoirs throughout the sequence of onshore and offshore Nile Delta^25^.

**Fig. 3:**
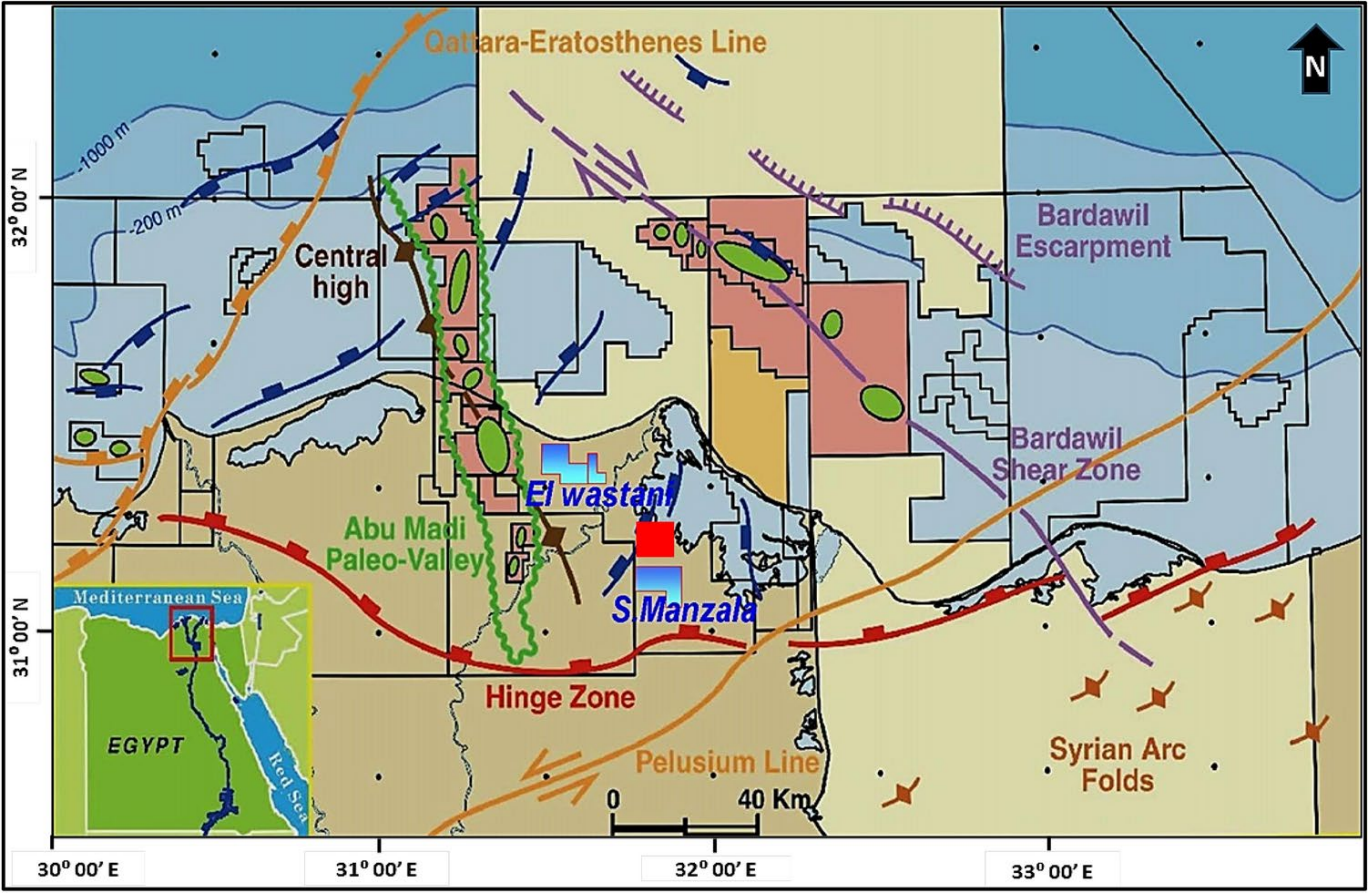
Schematic diagram illustrating the Main tectonic features of the Nile Delta and southeast Mediterranean region^26^. The green-colored areas refer to gas-producing fields, the blue-colored polygons refer to the main concession beside the study area; while the red-colored square refers to the study area, South Abu El Naga Field. Some arc folds are shown to the right down of the figure with a diagnostic symbol.

During the Messinian age, from approximately 7.24 to 5.33 million years ago, the Mediterranean Sea underwent significant geological changes, isolated from the Atlantic Ocean. This period, known as the Messinian salinity crisis, led to gypsum precipitation, salt formations, and the development of brackish water "Lago-Mare" facies^14,15^. The decline in sea levels resulted in widespread erosion, forming large canyons along the Mediterranean shorelines, and depositing extensive salt within the basin’s depocenter^14–16^. This crisis led to the development of major paleo-drainage systems along the northern coastline of Egypt, including the Nile Delta region, with canyons filled with deposits from the Qawasim and Abu Madi formations^15,16^. The study focuses on the Upper Messinian Abu Madi Formation, which occurs at depths of approximately 2600 to 3000 m and is a major gas reservoir in the Nile Delta. The Abu Madi Formation rests unconformably over the Qawasim Formation and is also unconformably overlain by the Kafr El Sheikh Formation^17,18^. It consists of cross-bedded sandstone and shale interbeds, with occasional conglomeratic levels (Fig. 2). The formation is the oldest gas producer in the region and is known for its good porosity, with an average of around 21%^18^. The onset of the Pliocene epoch brought a significant rise in sea level, flooding the entire Nile Delta basin and leading to the deposition of the Kafr El Sheikh Formation, characterized by thick marine shales and thin sandstone layers. Another abrupt drop in sea level during the Late Pliocene led to the deposition of the El Wastani Formation, representing a low-stand deltaic system^19^. The Abu Madi Formation is divided into two informal members: the upper Abu Madi Member and the lower Abu Madi Member^20,21^. The depositional origin of the formation is interpreted as fluvial to coastal marine, with sediments deposited in a subsiding basin influenced by a transgressive sea^21^. Determining the age of the Abu Madi Formation has been debated. While initially thought to be of the Early Pliocene age^20^. Recent studies have assigned it to the Late Miocene Messinian period based on rare benthonic foraminifera^20,21^. The formation serves as a reservoir for catagenetic gas sourced from deep hydrocarbon-generating source rocks, with some gas migrating upward to Pleistocene strata and shallower parts containing biogenic gas^19^. The South Abu El Naga Gas Field lies within the Nile Delta Basin, a region characterized by a complex geological history involving multiple tectonic events (Fig. 3). Here’s a summary focusing on the key events relevant to the geologic setting of the studied field:

- During the Jurassic and Early Cretaceous, the Nile Delta underwent east–west rifting, creating basins, followed by a period of thermal subsidence that formed a shelf margin^22^.
- Decades-long rifting gave way to a dramatic shift during the Late Cretaceous-Miocene. This tectonic squeeze folded former rift basins into the large northeast-southwest trending Syrian arc folds south of the Nile Delta^23,24^.
- The Nile Delta’s hinge line, an ancient shelf edge, separates thick Neogene sediments from the rifted margin, defining Egypt’s southern offshore limit^23^.
- The Nile Delta experienced a second round of rifting alongside the opening of the Gulf of Suez in the Oligo-Miocene. This northwesterly stretching reflects the separation of the Arabian and African plates^24^. It is also suggested that the pre-existing E-W and NW trending faults in the Nile Delta, possibly dating back to the Oligo-Miocene or earlier, might be linked to the initial stages of rifting in the Gulf of Suez^24^.

## Methodology and available data sets

This study investigates the reservoir potential of the Late Miocene Messinian gas-bearing heterogeneous sandstone reservoirs, in the South Abu El Naga Gas Field, Nile Delta, using integrated data derived from four wells SAEN-2, SAEN-4, SAEN-6, and SAEN-9 wells. Conventional triple combo well logs enabled petrophysical evaluation and estimating the main petrophysical parameters for reservoir potential prediction, while core data extracted from SAEN-2 well, comprising 35 core plugs from two cored intervals, was utilized in this study. This includes measurements of grain density (ρ_g_), helium porosity (∅_He_), permeability in horizontal and vertical directions (k_H_ and k_V_), as well as water saturation, in addition to the petrographic and mineralogical analyses. Collectively, these data contributed to a comprehensive reservoir characterization and identifying the hydraulic flow units. A detailed description of the applied technique has been published by many authors, and presented as follows.

### Petrographical techniques

In this sedimentological study, a set of petrographical techniques was utilized to examine the reservoir composition and its sedimentary structure. Two cored intervals were obtained extending for 11.5 m for the first interval and 7.0 m for the second interval with core recovery equals 86.95–96.42% (Table 1).

**Table 1:** The cored intervals in Abu Madi Formation in SAEN-2 well, the depth is estimated as true vertical depth subsea (TVDSS) and corrected for the depth shift.

Core number	Interval cut (m)	Interval recovered (m)	Recovered (m)	Core recovery (%)
1	2319.00–2330.50	2319.00–2329.00	10	86.95
2	2330.50–2337.50	2330.50–2337.25	6.75	96.42

Twenty-two cutting samples were chosen from the SAEN-2 well for in-deep petrographical, mineralogical, and morphological analysis within the depth interval of 2310–2329 m. These samples were impregnated with blue-dyed Araldite to describe the various pore types. The microfacies types were defined and nomenclated following the sandstone classification of Pettijohn et al.^28^. Additionally, thirteen representative samples, selected from ditch cuttings and cores, underwent back scattered scanning electron microscopy (SEM) analysis to describe the dominant the clay types, as well as the pore distribution and types. X-ray diffraction analysis (XRD) was also applied to determine the dominant clay minerals, where a semi-quantitative analysis was performed to establish the mineral composition of the isolated clay fraction (< 4 μm). Prior to further analysis, the samples were treatment with 35% hydrogen peroxide (H₃O₃) to eliminate any organic residues. The crushed samples were subsequently combined with purified water and subjected to ultrasonic vibrations in order to break down the clays. The clay fraction was extracted and then subjected to heating and glycolation in order to identify various minerals based on the XRD pattern^28–31^. A semi-quantitative examination was performed to ascertain the mineral composition of the isolated clay fraction, allowing for the identification of the prevailing clay types in the examined samples.

### Well-log techniques

The technique utilized conventional triple combo log measurements from the studied wells to assess petrophysical parameters like clay volume, porosity, and water saturation. Analyzing the caliper, gamma-ray, neutron, density, photoelectric effect factor (PEF), and resistivity measurements allowed for the identification of different clay types within the reservoir and comprehensive evaluation of the petrophysical characteristics using Techlog 2020.2 software.

Shale volume was estimated using two methods: a gamma-ray log for shale content calculation and a neutron-density approach for cross-plot analysis to determine the shale volume using the following formulas^32^:

1 $${\text{V}}_{{{\text{sh}}}} = \left( {{\text{GR}}_{{{\text{max}}}} - {\text{GR}}_{{{\text{log}}}} } \right)/\left( {{\text{GR}}_{{{\text{max}}}} - {\text{GR}}_{{{\text{min}}}} } \right)$$

2 $${\text{V}}_{{{\text{sh}}}} = \left( {\O_{{\text{N}}} - \O_{{\text{D}}} } \right)/\left( {\O_{{{\text{Nsh}}}} - \O_{{{\text{Dsh}}}} } \right)$$

Clay mineral types were identified through cross-plots using the (PEF) and spectral gamma-ray data (Th and K). Total porosity (Ø_T_) was calculated as the average of neutron and density porosities without accounting for shale volume. To adjust for shale volume impact, effective porosity (Ø_e_) was calculated as following:

3 $$\O_{{\text{T}}} = \surd \left( {\left( {\O_{{\text{N}}}^{{2}} {-}\O_{{\text{D}}}^{{2}} } \right)/{2}} \right)$$

For gas-bearing formations, the effective porosity (Ø_T_._gas_) was calculated using the corrected neutron and density porosities (Ø_NC_ and Ø_DC_) as following:

4 $$\O_{T.gas} = \sqrt {\frac{{\O_{N}^{2} + \O_{D}^{2} }}{2}}$$

5 $$\O_{e.gas} = \sqrt {\frac{{\O_{NC}^{2} + \O_{DC}^{2} }}{2}}$$

Calculating the net pay reservoir thickness required a knowledge on shale volume, effective porosity, and water saturation. The Indonesian model, commonly used for shaly sand interpretations to calculate the water saturation (Sw Indonesia) as follows^33^:

6 $${\text{Sw}}_{{{\text{Indonesia}}}} = \left\{ {\frac{{\sqrt {\frac{1}{{{\text{Rt}}}}} }}{{\left( {\frac{{{\text{Vsh}}^{{\left( {1 - 0.5{\text{Vsh}}} \right)}} }}{{\sqrt {{\text{Rsh}}} }}} \right) + \sqrt {\frac{{\O_{{\text{e}}}^{{\text{m}}} }}{{{\text{a}}.{\text{Rw}}}}} }}} \right\}^{{\left( {2/{\text{n}}} \right)}}$$

This model relates the deep resistivity to formation parameters such as formation water resistivity (Rw), shale resistivity (R_sh_), formation water saturation (Sw), and shale volume (V_sh_). To estimate the net pay thickness of the Abu Madi Formation in South Abu El Naga Field (SAEN), these values (Ø_e_ > 6%, V_sh_ < 35%, and Sw < 70%) were applied as cutoff values.

The Rw of the present reservoir has been estimated using Pickett plot, where the porosity is plotted on the Y-axis as a function of the LLD resistivity which is plotted on the X-axis, a set of Sw lines were constructed, and the intersection of the 100% Sw with the LLD resistivity with the 100% porosity is taken as Rw considering the lithology parameter (a) of Archie equals one.

### Conventional core data

A total of thirty-five samples were plugged from the Abu Madi reservoir in SAEN-2 well. They were cleaned using solvents in a Dean Stark apparatus and dried at 60 °C in a drying oven. The effective porosity of the cleaned plugs was measured by helium using a pycnometer, while the permeability values (k_V_ and k_H_) were estimated using a nitrogen permeameter.

The following equations were finally applied to estimate the flow zone indicator (FZI) and the reservoir quality index (RQI), utilizing core-derived permeability (k_H_), porosity (Ø_He_) and the normalized porosity index (Øz):

7 $$\O {\text{z}} = \O_{{{\text{He}}}} /\left( {{1} - \O_{{{\text{He}}}} } \right)$$

8 $$\left( {{\text{RQI}}} \right) = 0.0{314}*\left( {{\text{k}}_{{\text{H}}} /\O_{{{\text{He}}}} } \right)^{{0.{5}}}$$

9 $$\left( {{\text{FZI}}} \right) = {\text{RQI}}/\O {\text{z}}$$

where k_H_ is measured in mD, Ø_He_ and Øz are in decimals.

Also, the permeability anisotropy (λ_k_) is estimated as the ratio between the horizontal and vertical permeability values as follows^34^.

10 $${ \lambda }_{{\text{k}}} = \left( {{\text{k}}_{{\text{H}}} /{\text{k}}_{{\text{V}}} } \right)^{{{1}/{2}}}$$

The obtained conventional core data and the estimated reservoir quality parameters of the various reservoir rock types (RRTs) are listed in Table 2.

**Table 2:** The conventional core data in SAEN-2 well and the estimated flow zone and reservoir quality indices.

RRTs	Depth (m)	ρ_g_ (g/cm^3^)	k_H_ (mD)	k_V_ (mD)	λ_k_ (0.00)	∅_He_ (v/v)	Sw (%)	∅z (0.00)	RQI (μm)	FZI (μm)	R_35_ (μm)
RRT1	2324.5	2.66	16.01	0.73	4.68	0.18	68.6	0.22	0.18	0.82	2.268
	2326.2	2.69	2.4	NPP	–	0.12	57.0	0.136	0.14	1.027	1.055
	2326.8	2.68	2.76	NPP	–	0.12	70.5	0.136	0.15	1.10	1.145
	2324.3	2.68	27.5	1.53	4.24	0.20	62.8	0.25	0.37	1.48	2.846
	2321.8	2.68	86.3	7.67	3.35	0.21	62.7	0.266	0.64	2.408	5.345
	2323.8	2.67	86.5	1.0	9.30	0.20	63.4	0.25	0.65	2.60	5.583
	2322.8	2.67	263	NPP	–	0.24	54.9	0.316	1.03	3.262	9.171
	2320.3	2.65	429	25.9	4.07	0.26	55.9	0.351	1.28	3.643	11.412
	2326.3	2.69	367	NPP	–	0.24	57.0	0.316	1.22	3.863	11.156
	Average	2.674	142.27	7.37	5.13	0.20	61.42	0.249	0.629	2.245	5.553
	Min	2.650	2.40	0.73	3.35	0.12	54.90	0.136	0.140	0.820	1.055
	Max	2.690	429.0	25.90	9.30	0.26	70.50	0.351	1.280	3.863	11.412
RRT2	2320	2.64	325	13.0	5.00	0.22	60.8	0.282	1.20	4.255	11.198
	2319.8	2.67	659	7.89	9.14	0.26	53.0	0.351	1.60	4.554	14.688
	2326.5	2.69	416	NPP	–	0.22	59.5	0.282	1.37	4.857	12.947
	2323.3	2.65	474	3121	0.39	0.22	57.1	0.282	1.44	5.105	13.980
	2319.5	2.66	1250	2.98	20.48	0.27	56.9	0.37	2.16	5.84	20.715
	2324	2.67	2060	4.88	20.55	0.29	48.0	0.408	2.66	6.512	26.124
	2327.5	2.67	1064	13.5	8.88	0.26	56.7	0.351	2.01	5.721	19.467
	Average	2.664	892.6	527.2	10.74	0.25	56.00	0.332	1.777	5.263	17.017
	Min	2.640	325.0	2.98	0.390	0.22	48.00	0.282	1.200	4.255	11.198
	Max	2.690	2060	3121	20.55	0.29	60.80	0.408	2.660	6.512	26.124
RRT3	2320.8	2.67	1073	119	3.00	0.25	53.3	0.333	2.04	6.12	20.238
	2319.4	2.65	1385	618	1.50	0.26	50.7	0.351	2.28	6.489	22.732
	2321	2.67	829	4.64	13.37	0.23	58.6	0.299	1.88	6.294	18.689
	2321.3	2.67	1014	64.9	3.95	0.24	59.6	0.316	2.05	6.492	20.279
	2320.5	2.64	1643	83.6	4.43	0.26	52.0	0.351	2.52	7.172	25.134
	2325.5	2.68	1320	2273	0.76	0.25	55.0	0.333	2.30	6.9	22.860
	2325.8	2.69	2634	1788	1.21	0.29	48.8	0.408	2.90	7.1	30.186
	2327.3	2.67	2208	182	3.48	0.28	43.0	0.389	2.78	7.149	28.050
	2325	2.68	2947	1550	1.38	0.29	45.3	0.408	3.18	7.786	32.247
	2327.8	2.68	1696	4.86	18.68	0.25	52.7	0.333	2.6	7.8	26.490
	2328	2.67	2230	119	4.33	0.27	49.9	0.370	2.88	7.787	29.114
	2324.8	2.65	2751	1444	1.38	0.27	52.1	0.371	3.18	8.598	32.940
	2326	2.69	3120	2748	1.07	0.28	49.1	0.389	3.29	8.46	34.373
	Average	2.670	1911.5	846.1	4.50	0.26	51.55	0.358	2.606	7.242	26.410
	Min	2.640	829.0	4.64	0.76	0.23	43.00	0.299	1.880	6.120	18.689
	Max	2.690	3120	2748	18.68	0.29	59.60	0.408	3.290	8.598	34.373
RRT4	2321.5	2.67	2680	3.32	28.41	0.25	55.3	0.333	3.24	9.72	34.668
	2323	2.68	4377	NPP	–	0.28	49.8	0.389	3.95	10.157	41.944
	2325.3	2.67	2800	733	1.95	0.25	52.4	0.333	3.31	9.93	35.572
	2323.5	2.66	3642	3475	1.02	0.26	50.5	0.351	3.72	10.588	40.136
	2327	2.67	4893	1335	1.91	0.28	45.4	0.389	4.13	10.62	44.784
	Average	2.670	3678.4	1386.6	8.323	0.264	50.68	0.359	3.670	10.203	39.421
	Min	2.660	268	3.320	1.020	0.250	45.40	0.333	3.240	9.720	34.668
	Max	2.680	4893	3475	28.41	0.280	55.30	0.389	4.130	10.620	44.784

RRTs is the reservoir rock types, ρ_g_ is the grain density, k_H_ and k_V_ are the horizontal and vertical permeabilities, respectively, ∅_He_ is the helium porosity in fraction, NPP means not possible plug, λ_k_ is the permeability anisotropy, Sw is the water saturation, ∅z is the normalized porosity index, RQI and FZI are the reservoir quality and flow zone indices, respectively, R_35_ is the effective pore radius.

The water saturation (Sw) is estimated using the retort oven method which can estimate the Sw as a complementary value for the gas saturation. The water is extracted and estimated by heating and evaporation at 105 °C.

As a reservoir quality parameter, the effective pore radius (R_35_) is defined as the radius of the pore space that corresponds to filling with 35% of the total mercury volume that is injected into the rock sample in a mercury injection capillary pressure test. It has been estimated following Winland as follows^13^.

11 $${\text{R}}_{{35}} = 10^{{0.732 + 0.588\log {\text{K}}_{{\text{H}}} - 0.864\log \O _{{{\text{He}}}} }}$$

where k_H_ is the air permeability in mD, and Ø is the porosity in %.

## Results and discussion

**Fig. 4:**
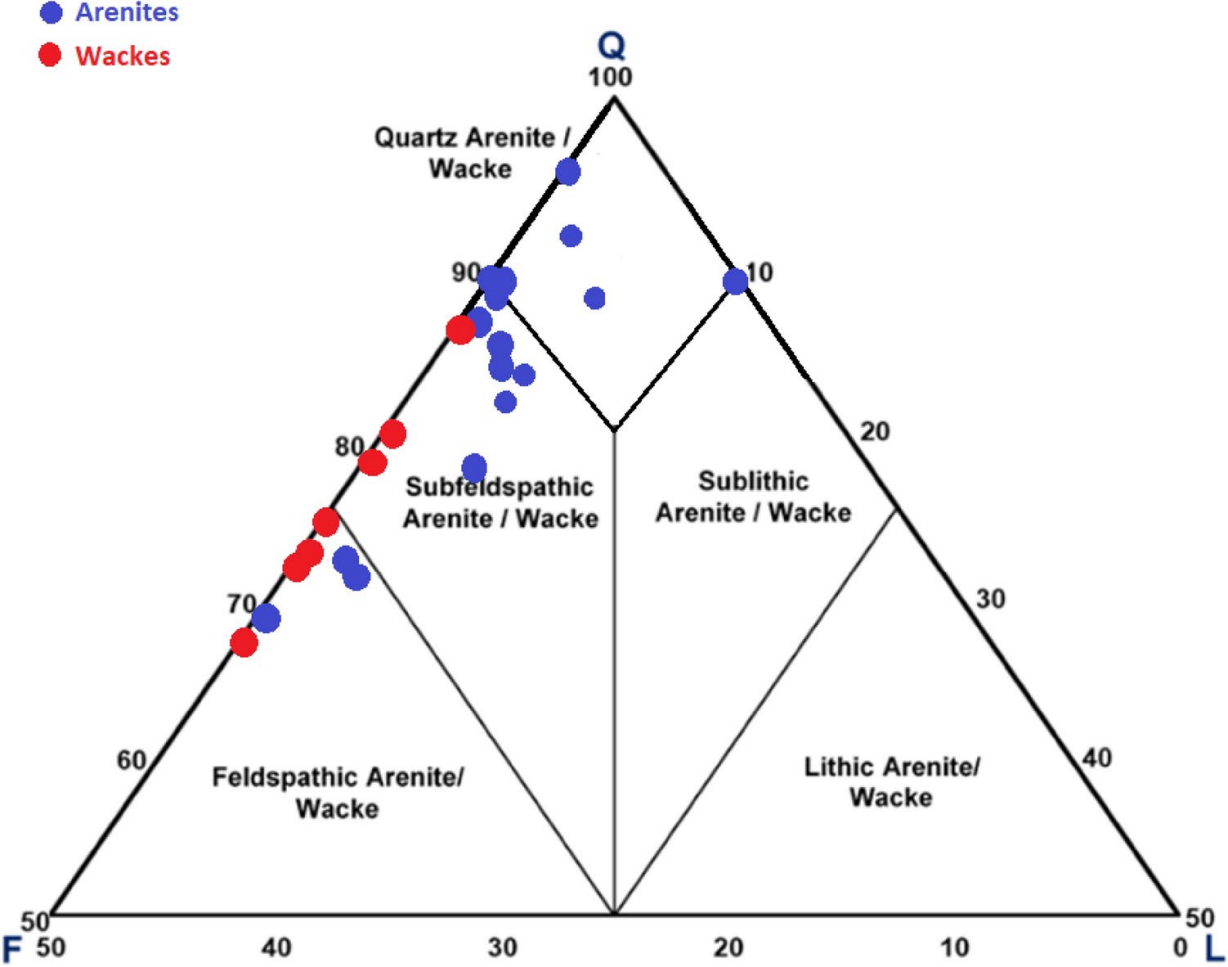
Ternary diagram (QFL) of Pettijohn et al.^18^ showing the framework compositions and classification of the Abu Madi Formation.

The clastic composition of the Abu Madi sequence was matched using the QFL ternary diagram of Pettijohn et al.^18^. Therefore, it is classified into quartz arenite, subfeldspathic quartz arenite, subfeldspathic wackes, feldspathic quartz arenite, and feldspathic wackes (Fig. 4).

### Petrographic description

**Fig. 5:**
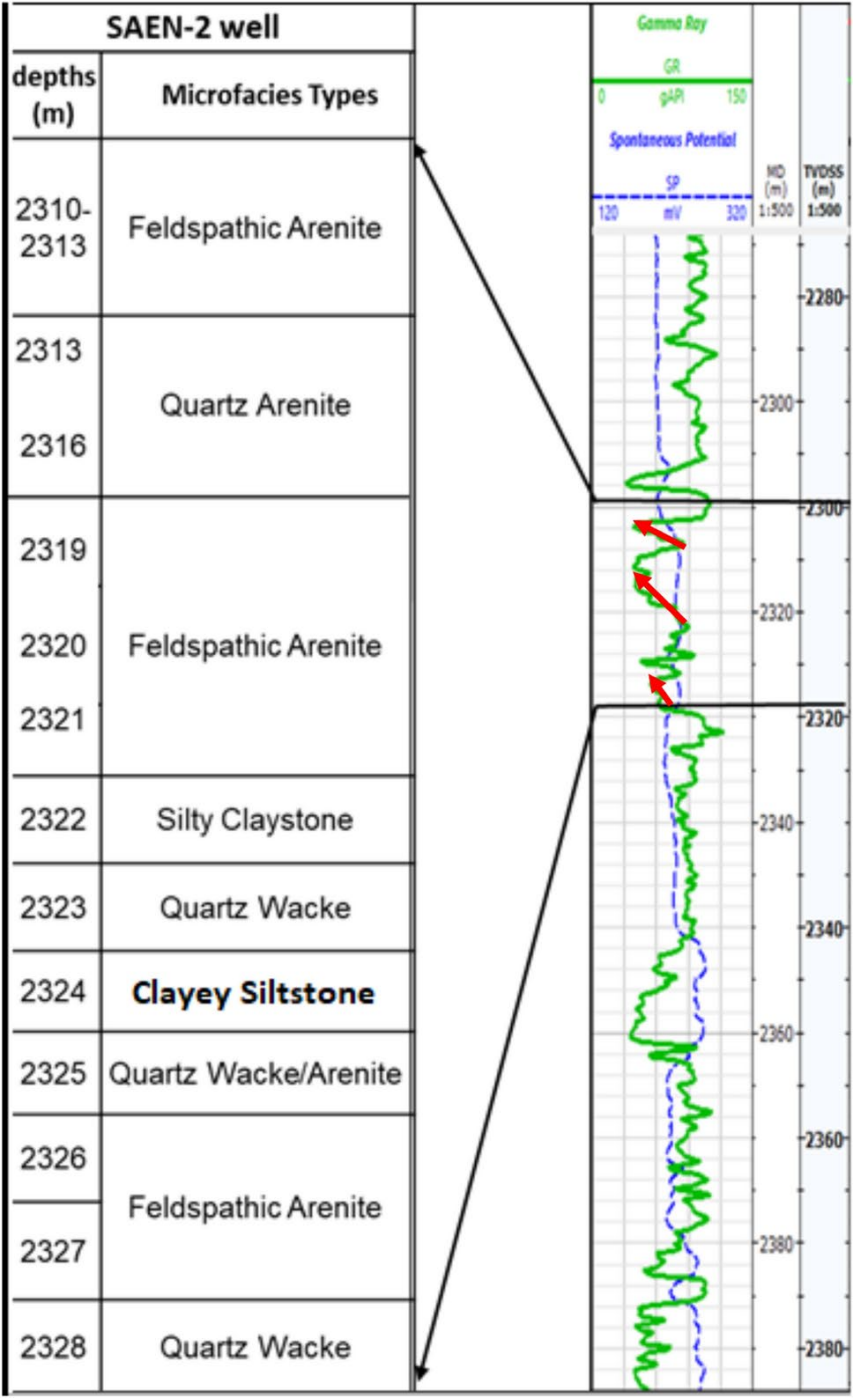
Microfacies types of the Abu Madi Formation in the SAEN-2 well accompanied with their corresponding gamma-ray values. For more details on the microfacies description see Fig. 6.

**Fig. 6:**
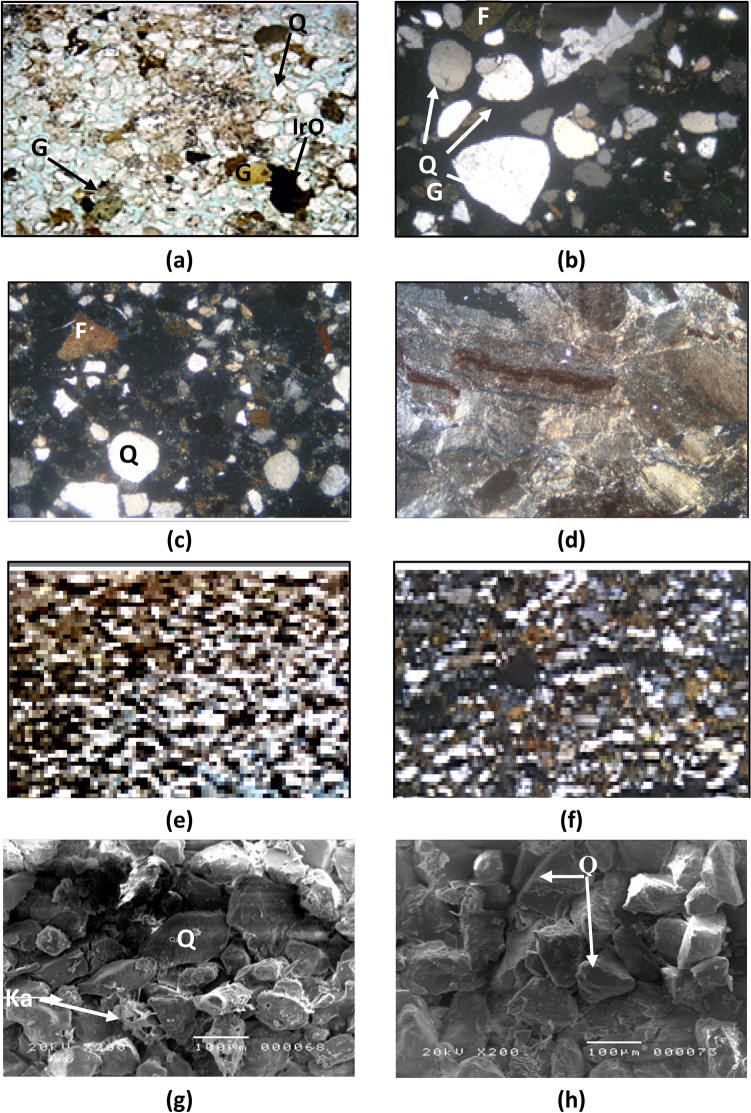
Photomicrographs of the Abu Madi microfacies in the SAEN-2 well showing (**a**) very good intergranular porosity of 35% (dyed-blue), abundant monocrystalline quartz grains quartz grains (Q), iron oxides (IrO), and glauconite (G), 10X, PPL, feldspathic arenite, (**b**) poorly-sorted quartz grains with abundant quartz grains, feldspars, and silt-sized glauconite, 20X, XPL, quartz arenites, (**c**) poorly sorted quartz grains with a few feldspar grains, 20X, XPL, quartz arenite, (**d**) silty-sized quartz grains embedded in claystone matrix, 40X, PPL, feldspathic wacke, (**e**) silty-sized quartz grains embedded in much clays with some micro intergranular pore spaces, 20X, PPL, clayey-siltstone, (**f**) many silt-sized quartz grains and a few feldspars, embedded in clay matrix, and aligned with some very thin shale streaks microfacies, 20X, XPL, quartz wacke/arenite microfacies (**g**) some kaolinite booklets (Ka) filling the pore spaces of the feldspathic arenite, and (**h**) some micrite crystals filling some the pore spaces (M) in between some quartz grains, feldspathic arenite.

The described microfacies of the Abu Madi Formation in the SAEN-2 well are illustrated in Figs. 5 and 6, which is accompanied by the gamma-ray values. The Abu Madi sequence in the SAEN-2 well seems to be repeated in a set of coarsening upward cyclicity, where it starts with quartz wacke/arenite at the base (2328 m) changes upward into felspathic and quartz arenites. Then the cycle is repeated again with some quartz wacke/arenite, feldspathic wacke, and subfeldspathic wacke, at depth interval (2322–2325 m), and coarsening upward changing into feldspathic arenite at the top of the cored depth interval (2310–2313 m). This coarsening upward cyclicity indicates the deposition in a prograding shoreface and deltaic environments. This sedimentological structure setting is accompanied by decreasing the gamma-ray values upward in a cyclicity coincident with the coarsening upward cyclicity (Fig. 5). A detailed description of the studied sequence and its microfacies has been published by Khalifa et al.^34^. This sequence is primarily composed of feldspathic arenite, quartz arenite, feldspathic wacke, subfeldspathic wacke, and quartz wacke/arenite microfacies which are described as follows.

#### Quartz wacke/arenite

The microfacies is identified at a depth interval of 2328–2329 m (Fig. 6f). It exhibits a good reservoir quality with a visible porosity of 15–20% and accounts for 9.1% of the Abu Madi sample in the reservoir under investigation. The composition consists of silt-sized, subangular to subrounded, poorly-sorted, loose, monocrystalline quartz grains that display a moderately compacted structure with point and straight grain-to-grain contacts. Recorded are rare K-feldspars and plagioclase, opaques, glauconite, rock fragments, and iron oxides. An abundance of clays cements the detrital grains at depths of 2323 m and 2328 m, with a thickness of 2.0 m (Fig. 6f). Porosity of this microfacies may be considered fair (10–15%) and is mostly attributed by intergranular and matrix porosity.

#### Subfeldspathic wacke (clayey siltstone)

This particular microfacies accounts for a small proportion of the reservoir examined, specifically 9.1% of the Abu Madi sample. It consists of monocrystalline quartz grains of silt size, which exhibit straight extinction (Fig. 6e). Identification of K-feldspars and plagioclase, as well as smectite and iron oxides, has been made at a depth of 2324 m and a thickness of 1.0 m. Although the argillaceous nature of this microfacies it still has some intergranular micro pore spaces (dyed-blue, Fig. 6e).

#### Feldspathic wacke (silty claystone)

This microfacies is located in the central section of the Abu Madi Formation and accounts for a small proportion of the research reservoir, specifically 9.1% of the Abu Madi sample. It consists of quartz grains that are silt-sized, monocrystalline, and straight-extinction. The rock deposit consists of K-feldspars, plagioclase, and iron oxides, located at a depth of 2322 m and having a thickness of one meter (Fig. 6d). XRD analysis has identified that the detrital grains are bound together by smectite and kaolinite clays. The rock is embedded in a claystone matrix, which is fairly compacted and fissile. The clay phase consists mostly of smectite and kaolinite minerals, which are characterized by flaky form structures.

#### Quartz arenite

Profusion of monocrystalline poorly-sorted subrounded quartz grains exhibiting straight extinction. Seldom occurring K-feldspars and plagioclase, with a very fine to fine-grained (average fine) texture, ranging from subangular to subrounded, poorly-sorted, and loosely packed (Fig. 6b). It constitutes about 13.6% of the total prepared samples. The reservoir exhibits good quality, with a very good visual intergranular porosity and meso vugs up to 25–30% due to intensive dissolution, this microfacies is represented in depth interval 2313–2316 m, indicating 3m thickness (Fig. 6c).

#### Feldspathic arenite

This microfacies occurs in majority of the analyzed samples (59.1% of the Abu Madi samples). It consists subangular to subrounded fairly sorted quartz grains, with some feldspar grains represented in depth intervals 2310–2313 m, 2319–2321 m and 2326–2327 m. This microfacies shows 8 m as a total thickness (Fig. 6a). The feldspar grains increase in percentage and change into feldspathic arenite microfacies streaks in the vertical direction through the core. In addition, a few foraminiferal tests, and lithic micritic fragments are noticed. These components are incorporated in clayey and micritic matrix. Partly leached framework feldspar grains and mica flakes are locally found. Minor distributed pore filling and grain coating detrital clay matrix and poorly-developed euhedral quartz overgrowths are recorded. Quartz overgrowth aided slightly in minimizing the pores. However, the visual porosity is still quite good and represented by intergranular, vuggy, and micro channel porosity, due to dissolution and leaching out, sometimes these pore spaces are partly filled by vermicular clays. Generally, it is characterized by good to very good visual porosity ranging between 25 and 30% (Fig. 6a).

### Scanning electron microscopy (SEM)

The studied samples are mainly composed of quartz grains cemented by silica with a few calcite and clay patches. In certain subfeldspathic arenites with a few calcareous cement as indicated from the XRD analysis (2–10%, Table 3), and silt-sized siderite grains occupy some pores, suggesting the formation of authigenic minerals. Intergranular pores predominate, though kaolinite booklets fill some pores in the subfeldspathic greywacke microfacies. Despite the presence of kaolinite, porosity remains effective in these samples.

**Table 3:** Semi-quantitative relative abundance of the mineralogic composition of the studied Abu Madi microfacies in SAEN-2 well in South Abu El Naga Gas Field, the mentioned depth is a true vertical depth subsea (TVDSS).

Depth (m)	Microfacies types	Quartz (%)	Feldspar (%)	Lithics (%)	Smectite (%)	Illite (%)	Kaolinite (%)
2304	Subfeldspathic arenite	77	7	5	5	–	6.1
2307	Subfeldspathic arenite	78	5	5	6.8	–	5.2
2310	Quartz arenite	87	3	5	3	–	2
2313	Quartz arenite	89	3	3	3	–	2
2316	Quartz arenite	90	–	10	–	–	–
2319	Feldspathic arenite	71	19	3	5.6	–	1.4
2320	Feldspathic arenite	70	19	3	6	–	2
2321	Feldspathic arenite	74	14	2	3	1	6
2322	Subfeldspathic wacke	35	5	0	39	–	21
2323	Quartz arenite	76	7	4	9.1	–	3.9
2324	Subfeldspathic wacke	70	15	–	9.8	–	5.3
2325	Quartz Arenite	83	5	4	5	–	3
2326	Feldspathic arenite	84	3	–	4.9	3.1	4.9
2327	Feldspathic arenite	83	8	–	5.4	–	3.6
2328	Quartz arenite	79	9	–	4.3	3.4	4.3
2330	Subfeldspathic wacke	43	20	–	20.7	–	16.3
2331	Feldspathic arenite	64	20	–	8	–	8
2332	Feldspathic arenite	61	25	–	7	–	7
2333	Feldspathic arenite	63	20	–	9	–	8
2334	Feldspathic arenite	60	21	–	10.6	–	8.4
2335	Quartz wacke/arenite	60	15	–	15	–	10
2336	Quartz Arenite	80	10	–	6	–	4

### X-Ray diffraction analysis (XRD) and clay mineralogy

X-ray diffraction (XRD) analysis was performed on the twenty-two samples of the SAEN-2 well to identify the clay mineral types in the Abu Madi Formation. The XRD measurements show a composition of quartz, feldspars, calcite, kaolinite, and smectite in the samples. The Abu Madi microfacies consist mainly of feldspathic arenites, quartz wacke/arenites, subfeldspathic wacke, and clayey siltstone. Sand grains are primarily composed of quartz (60–90%), with lesser amount of feldspar, calcite, smectite, illite, and kaolinite (Table 3). Most samples have clay mineral content (smectite and illite) below 20%, except for higher levels of clay minerals found at certain depths. Smectite and kaolinite are the dominant clay types, they increase with increasing depth and possibly resulted from feldspar alteration.

### Conventional core data

#### Rock typing based on core data

**Fig. 7:**
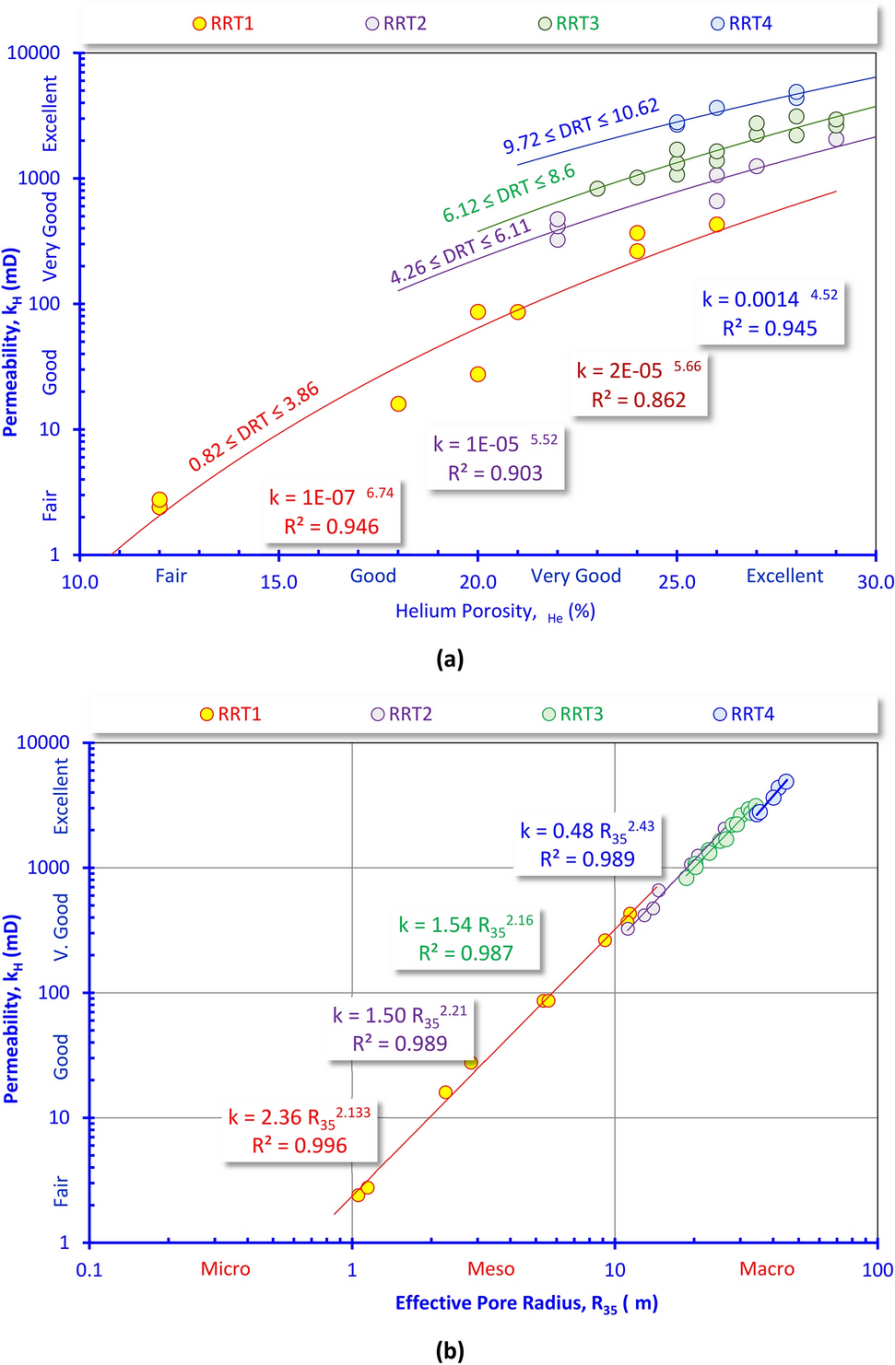
Rock typing based on (**a**) the horizontal permeability (k_H_)-porosity, and (**b**) the permeability-R_35_ plots for the core data of the Abu Madi Formation.

**Fig. 8:**
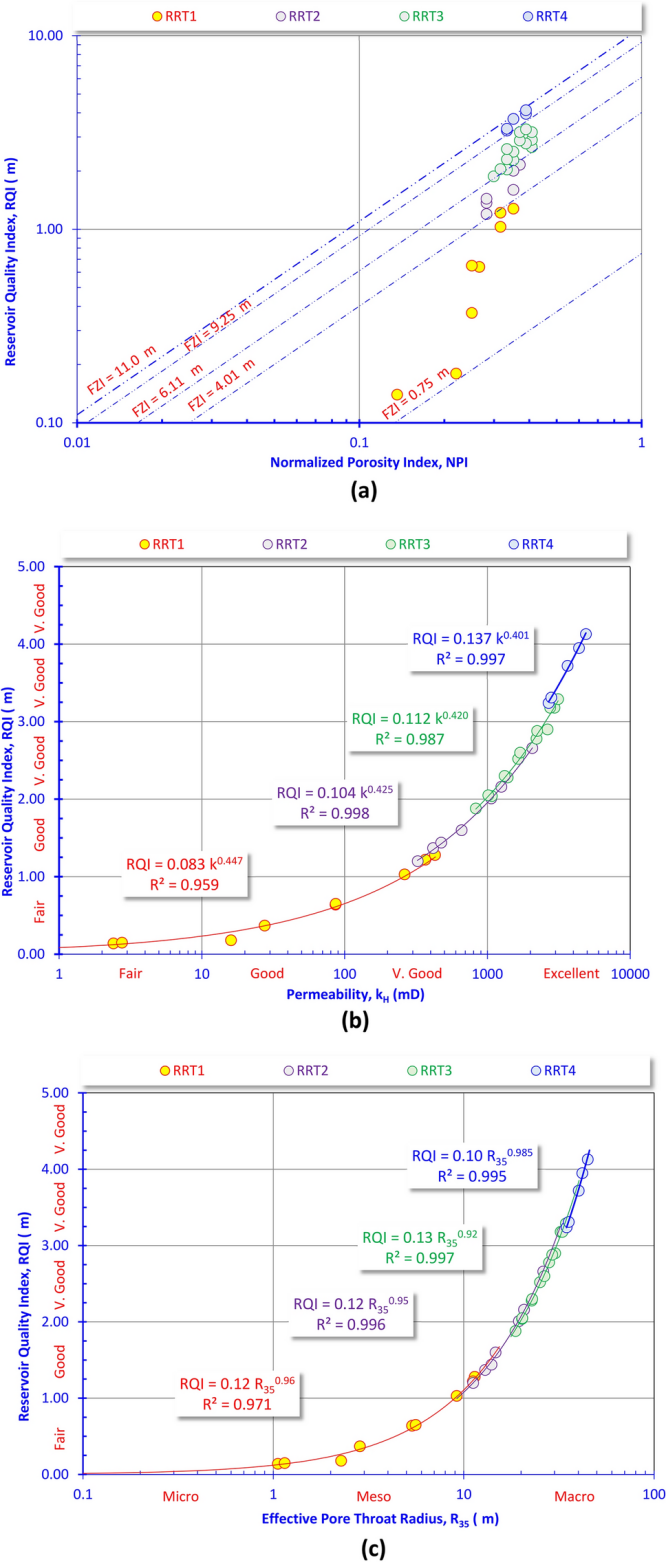
Rock typing based on (**a**) the RQI-NPI plot, (**b**) the RQI-permeability (k_H_) plot, and (**c**) the RQI-R_35_ plot for the core data of the Abu Madi Fm.

This study integrates the core analysis, petrography, and well log data to characterize the reservoir and identify the RRTs in the Abu Madi reservoir in the Nile Delta Basin. Rock typing and discriminating the reservoir into distinct (RRTs) based on the permeability–porosity plot (k-∅, Fig. 7a), permeability-pore throat sizes (k-R_35_, Fig. 7b) plot, and the RQI-NPI plot supported by the FZI values (Fig. 8a), which is essential for building realistic reservoir models^34–40^. Indeed, the discrimination of flow units and reservoir rock types relies on the fluid flow ability through the reservoir represented by the flow zone indicator (FZI) and reservoir quality index (RQI), that are considered the main reservoir quality key parameters that are widely used in reservoir simulation^41–44^.

Plotting the permeability versus porosity indicates the presence of four petrophysical/fluid flow properties/behaviors represented by four RRTs, all of them are characterized by fair to excellent porosity and permeability values, i.e., all are considered promising for storing and delivering fluids (Fig. 7a). These four RRTs are characterized by meso to macro pore sizes (1.0 μm < R_35_ < 100 μm) (Fig. 7b). This plot may be not helpful in rock typing but it helps in explaining the excellent quality of the Abu Madi reservoir zones. It indicates that the high reservoir quality of the RRT2-4 is due to having macro pores (Fig. 7b, 10 μm < R_35_ < 100 μm following the classification of Nabawy et al.^37,38^ of high connectivity.

The RQI-NPI plot superimposed by the FZI values indicates that the Abu Madi samples could be subdivided into four RRTs with the best quality assigned to the RRT4, while the RRT1 has fair reservoir quality as follows.

RRT1 is characterized by fair to excellent porosity and dominance of meso pore spaces (1.0 μm < R_35_ < 10 μm, Fig. 7b) causing a fair to very good horizontal permeability (Fig. 7a). These reservoir properties caused a poor to fair FZI values (0.75 μm < FZI < 4.01 μm, Fig. 8a, Table 2), i.e., fair reservoir quality, following the classification of Nabawy et al.^37–42^. Petrographically, it is composed of fairly to highly porous feldspathic wacke, and subfeldspathic wacke microfacies but with many intercalations with highly porous quartz arenite-dominated thin laminas (Fig. 6).

RRT2 samples have very good to excellent porosity and dominance of macro pore spaces (10 μm < R_35_ < 100 μm, Fig. 7b) causing mostly a very good permeability (k_H_ > 325 mD, Fig. 7a). These reservoir properties caused a good FZI values (4.01 μm < FZI < 6.11 μm, Fig. 8a, Table 2), i.e., good reservoir quality is assigned. These samples are primarily composed of relatively highly porous feldspathic wacke, subfeldspathic wacke, quartz wacke/arenite and quartz arenite microfacies (Fig. 6).

The RRT3 type is dominated by macro pore spaces (10 μm < R_35_ < 100 μm, Fig. 7b) and very good to excellent porosity causing a very good to excellent permeability (k_H_ > 829 mD, Fig. 7a). These reservoir properties caused a good FZI values (6.11 μm < FZI < 9.25 μm, Fig. 8a, Table 2), i.e., good reservoir quality is assigned for this rock type. It consists of highly porous feldspathic arenite and quartz arenite microfacies in addition to some quartz wacke/arenite microfacies content.

The RRT4 has the best reservoir properties in Abu Madi reservoir, it is predominated by macro pore spaces (10 μm < R_35_ < 100 μm, Fig. 7b) and excellent porosity values (25% ≤ ∅, Fig. 7a) contributing to excellent permeability (k_H_ > 2680 mD, Fig. 7a). These reservoir properties caused a very good FZI values (8.25 μm < FZI < 11.0 μm, Fig. 8a, Table 2), i.e., very good reservoir quality is assigned for this rock type. It is mostly predominated by highly porous feldspathic and quartz arenites of intergranular to vuggy pore spaces due to dissolution and leaching out (Fig. 6).

Considering the permeability anisotropy (λ_k_) which varies between 0.39 and 28.41 (Table 2), it indicates highly to extremely highly anisotropic primary fabrics (λ_k_ > 4.0) due to the presence of many shale streaks in most plug samples, especially RRT1 and RRT2 in spite of presence of one extremely highly anisotropic sample in the RRT4 samples.

Finally, the water saturation of the RRT1 samples fluctuates between 54.9 and 70.5% (Table 2), mostly due to the presence of some scattered authigenic clays as indicated from the petrography of the feldspathic wacke, subfeldspathic wacke, and quartz wacke/arenite composition (Fig. 6), while for the other RRTs samples have Sw less than 60.8% (Table 2), due to less clay content as indicated from the their dominant feldspathic and quartz arenite composition (Fig. 6).

#### Reservoir quality attributes

As aforementioned in Fig. 8a–c, the reservoir quality, represented by the RQI and FZI values, is primarily influenced by the horizontal permeability (k_H_) and effective pore radius (R_35_). The reservoir quality serves as an indicator of the reservoir’s capacity to deliver fluid content corresponding to a specific porosity value. In summary, for two given reservoirs with identical porosity but differing permeabilities, a higher permeability value indicates a greater capacity of the reservoir’s porosity to transmit fluids, i.e., signifying superior reservoir quality.

Plotting the horizontal permeability as a function of both the helium porosity and R_35_ (Fig. 8a and b) indicates that the R_35_ is the main attribute of the permeability (k_H_), while the porosity is an additional attribute depending on the type and aperture of the pore spaces, and their ability to contribute to the R_35_, and the permeability as well.

Thereby, the permeability (k_H_) and the R_35_ are the main contributors to the reservoir quality (RQI) of various microfacies of the Abu Madi reservoir as indicated from the RQI-k (Fig. 8b) and the RQI-R_35_ (Fig. 8c) plots. The high R_35_ values of the studied RRTs are mostly attributed to the dominance of the intergranular pore spaces feldspathic, quartz arenite, and the subfeldspathic wacke microfacies, in addition to the dominance of vuggy pore spaces, due to dissolution and leaching out, and matrix porosity of the feldspathic arenite, quartz wacke/arenite, and the feldspathic wacke to subfeldspathic wacke microfacies (Fig. 6).

As an output of the present study and due to the reliable interrelationships between the various petrophysical parameters, a set of highly reliable mathematical models can be applied to estimate the horizontal permeability (k_H_), the effective pore radius and the reservoir quality index in terms of each other as follows.

**Table:** 

RRT1	k_H_ = 10^–7^ ∅_He_^6.74^	(R^2^ = 0.946)
	k_H_ = 2.36 R_35_^2.13^	(R^2^ = 0.996)
	RQI = 0.083 k_H_^0.44^	(R^2^ = 0.959)
	RQI = 0.12 R_35_^0.96^	(R^2^ = 0.971)
RRT2	k_H_ = 10^–5^ ∅_He_^5.52^	(R^2^ = 0.903)
	k_H_ = 1.50 R_35_^2.21^	(R^2^ = 0.989)
	RQI = 0.104 k_H_^0.43^	(R^2^ = 0.998)
	RQI = 0.12 R_35_^0.95^	(R^2^ = 0.996)
RRT3	k_H_ = 2 × 10^–5^ ∅_He_^5.66^	(R^2^ = 0.862)
	k_H_ = 1.54 R_35_^2.16^	(R^2^ = 0.987)
	RQI = 0.112 k_H_^0.42^	(R^2^ = 0.987)
	RQI = 0.13 R_35_^0.92^	(R^2^ = 0.997)
RRT4	k_H_ = 0.0014 x ∅_He_^4.52^	(R^2^ = 0.945)
	k_H_ = 0.48 R_35_^2.43^	(R^2^ = 0.989)
	RQI = 0.137 k_H_^0.40^	(R^2^ = 0.997)
	RQI = 0.10 R_35_^0.99^	(R^2^ = 0.995)

From this mathematical model, the horizontal permeability (k_H_) is exponentially related to the porosity and R_35_ with a very high reliability (R^2^ ≥ 0.862 & 0.987, respectively). For the k_H_-∅_He_ model, the multiplication factor and the exponent increase with increasing the reservoir quality from the RRT1 to the RRT4.

For the k-R_35_ model, the equation exponent increases, in general, with increasing the reservoir quality, while the multiplication factor decreases, i.e., increasing the exponent values of both the k-∅ and the k-R_35_ models is an indication for increasing the reservoir quality as mentioned by many authors^43–47^.

On the other hand, the RQI is related to the horizontal permeability (k_H_) and the effective pore radius in highly reliable exponential relationships (R^2^ ≥ 959). In general, the RQI-k exponent is mostly constant (0.42–0.44) and similarly the RQI-k multiplication factor is also constant (0.10–0.13) as referred by many authors^48–50^, while the RQI-k multiplication factor and the RQI-R_35_ exponents increase with increasing the reservoir quality. Thereby, increasing the RQI-k multiplication factor and the exponent values of the k-∅, the k-R_35_, and the RQI-R_35_ are indicators for increasing the pore throat radius and connectivity, i.e., they are considered indicators for the dominance of intergranular, micro channels, and interconnected vuggy, due to dissolution, pores which is supported by the petrographical study (Fig. 6). This dissolution origin is in accordance with many literature that referred to the dissolution origin for both channels and vugs^51–55^.

### Reservoir quality and lithology identification

Techniques like mud logging and well logging aid in the interpretation of the lithology of subsurface formations during borehole drilling operations. The well logging technique is a crucial method for gathering geological data, enabling comprehensive subsurface geological and petrophysical analysis. The interpretation of well logging data includes both qualitative approaches like cross-plot analysis and lithologic description, as well as quantitative methods such as petrophysical evaluation. These integrated methodologies offer an extensive comprehension of the subsurface lithology of the Abu Madi Formation.

#### Cross-plot technique

**Fig. 9:**
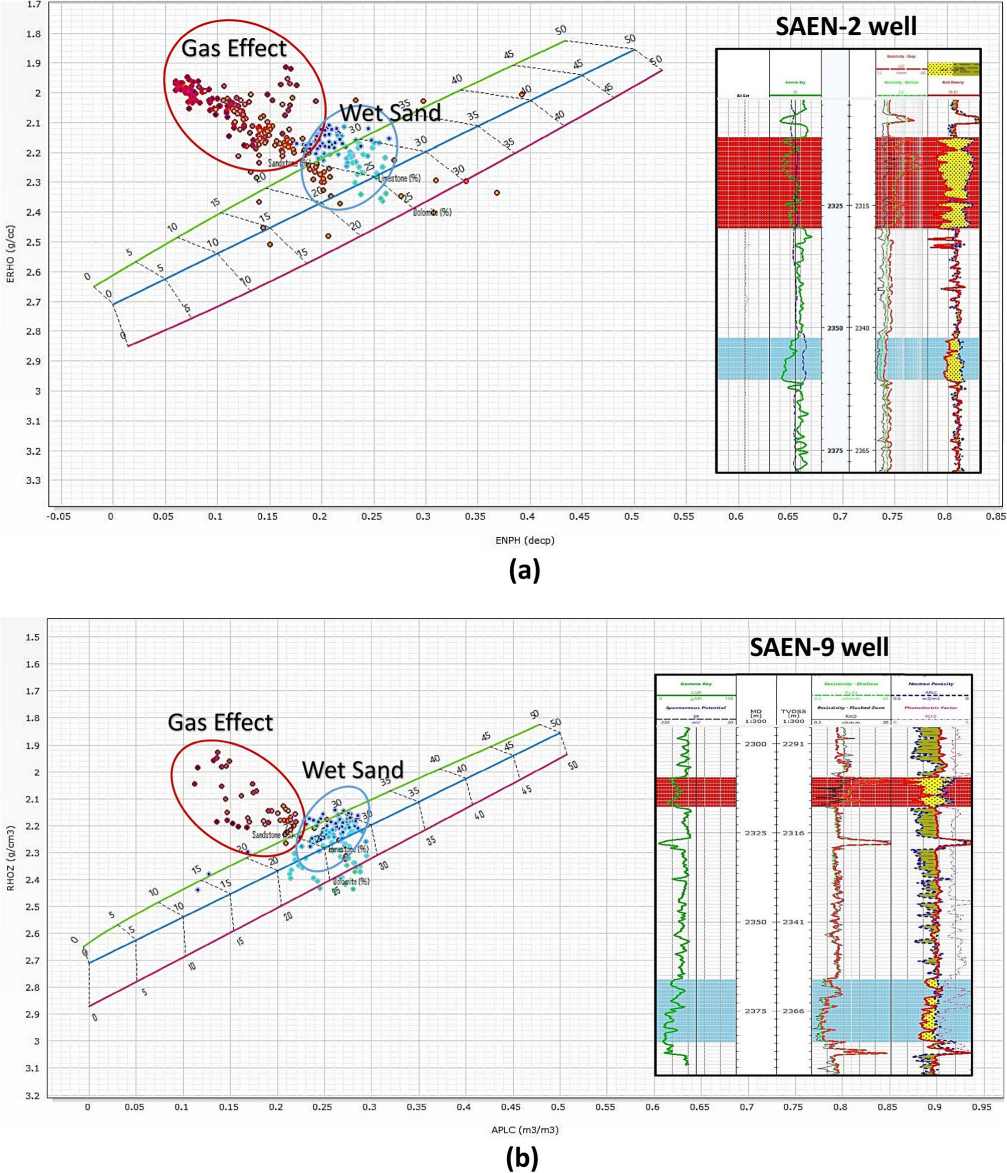
X–Y plot correlation between the neutron (APLC) & bulk density (RHOZ) of the Abu Madi pay zones and wet zone intervals in SAEN-2 (**a**) and SAEN-9 (**b**) wells. The first and second tracks of the triple combo to the right of the figures represent the depth, caliper, and gamma-ray, logs, while the third track represents the resistivity logs, and the last track to the right represent the density-neutron logs.

The cross-plot techniques are utilized to evaluate electric log data from wells SAEN-2 and SAEN-9, as examples of the studied wells, aiming to identify lithology and determine reservoir parameters. The neutron-density cross-plot is particularly effective for identifying the lithology of the Late Miocene Messinian reservoir, revealing a predominance of sandstone as indicated from the petrographical study (Fig. 6), with some interspersed shale and shifted points presented by red circles indicating the gas implication in the Abu Madi Formation within the SAEN Field. To check the gas effect in the studied pay zones, the well log data are presented on a set of cross-plots. The presented pay zones in the RHOZ-NPHI cross-plots are presented by red circles shifted up due to the gas effect (Fig. 9a, b) and highlighted red for the upper zone and blue for the lower zone of the Abu Madi reservoir sequence in the triple combo plot to the right of the plots (Fig. 9a, b).

#### Clay types

**Fig. 10:**
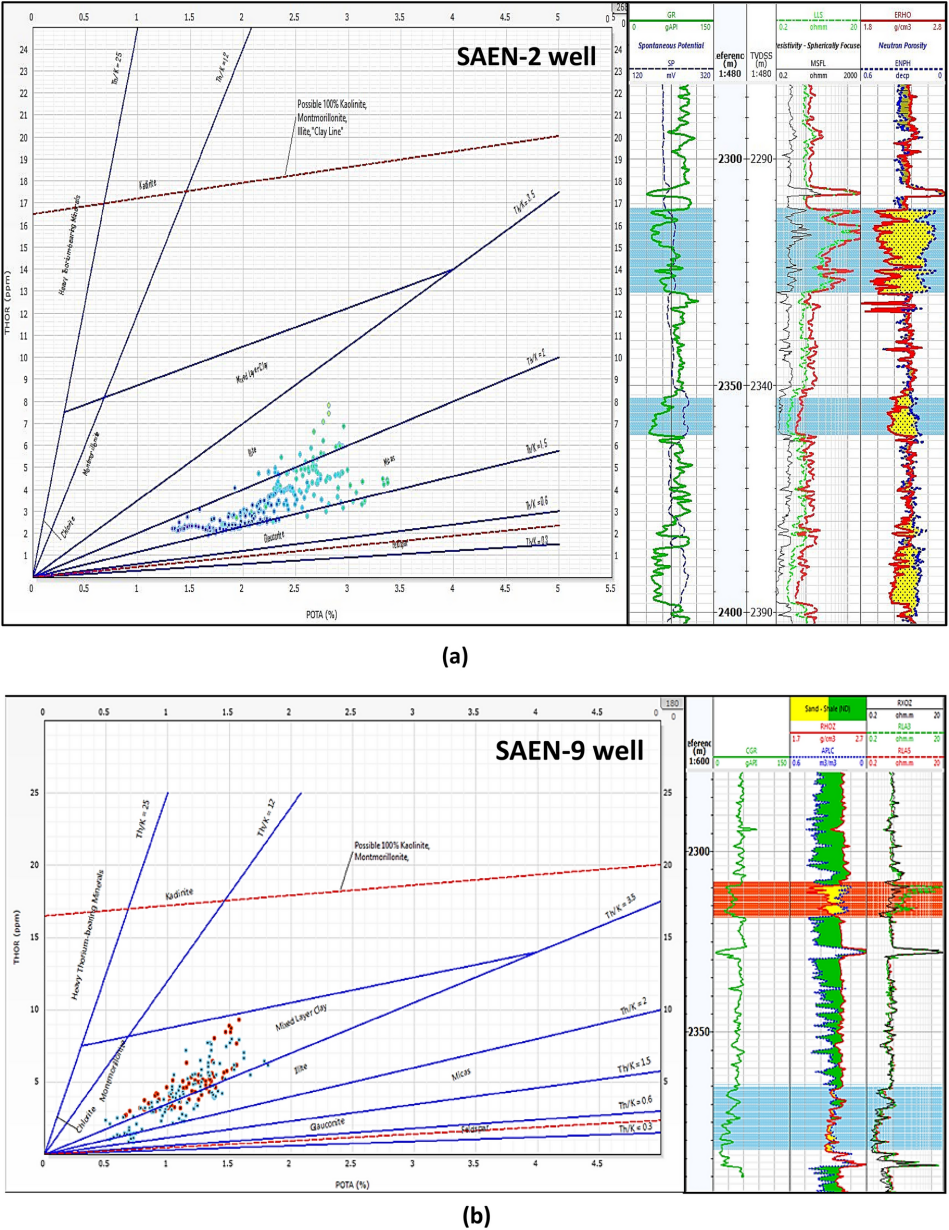
X–Y plot between thorium & potassium illustrating the clay minerals types of the Abu Madi Formation in (**a**) SAEN-2 well, and (**b**) SAEN-9 well.

**Fig. 11:**
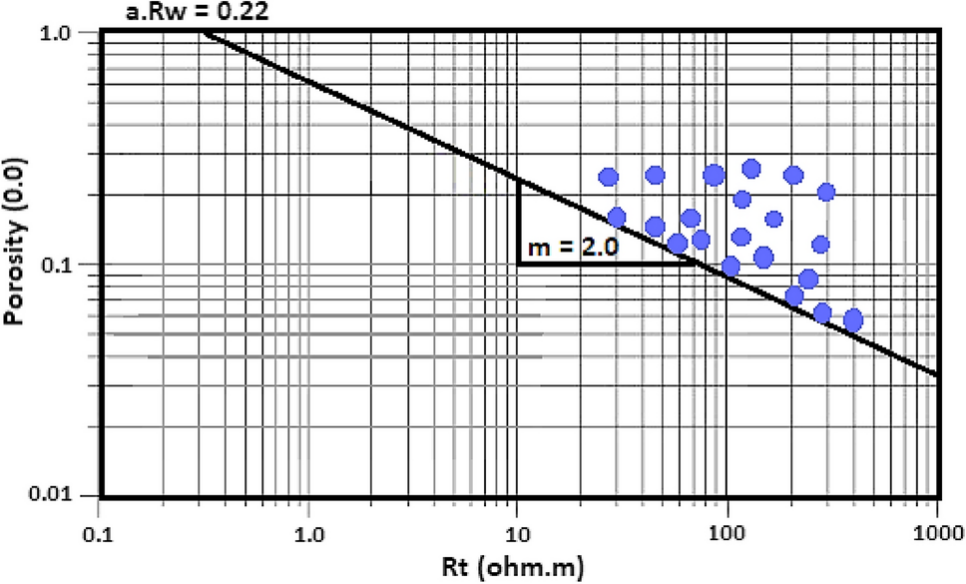
Pickett plot of the Abu Madi sequence indicating that Rw = 0.22 ohmm considering a = 1.

**Fig. 12:**
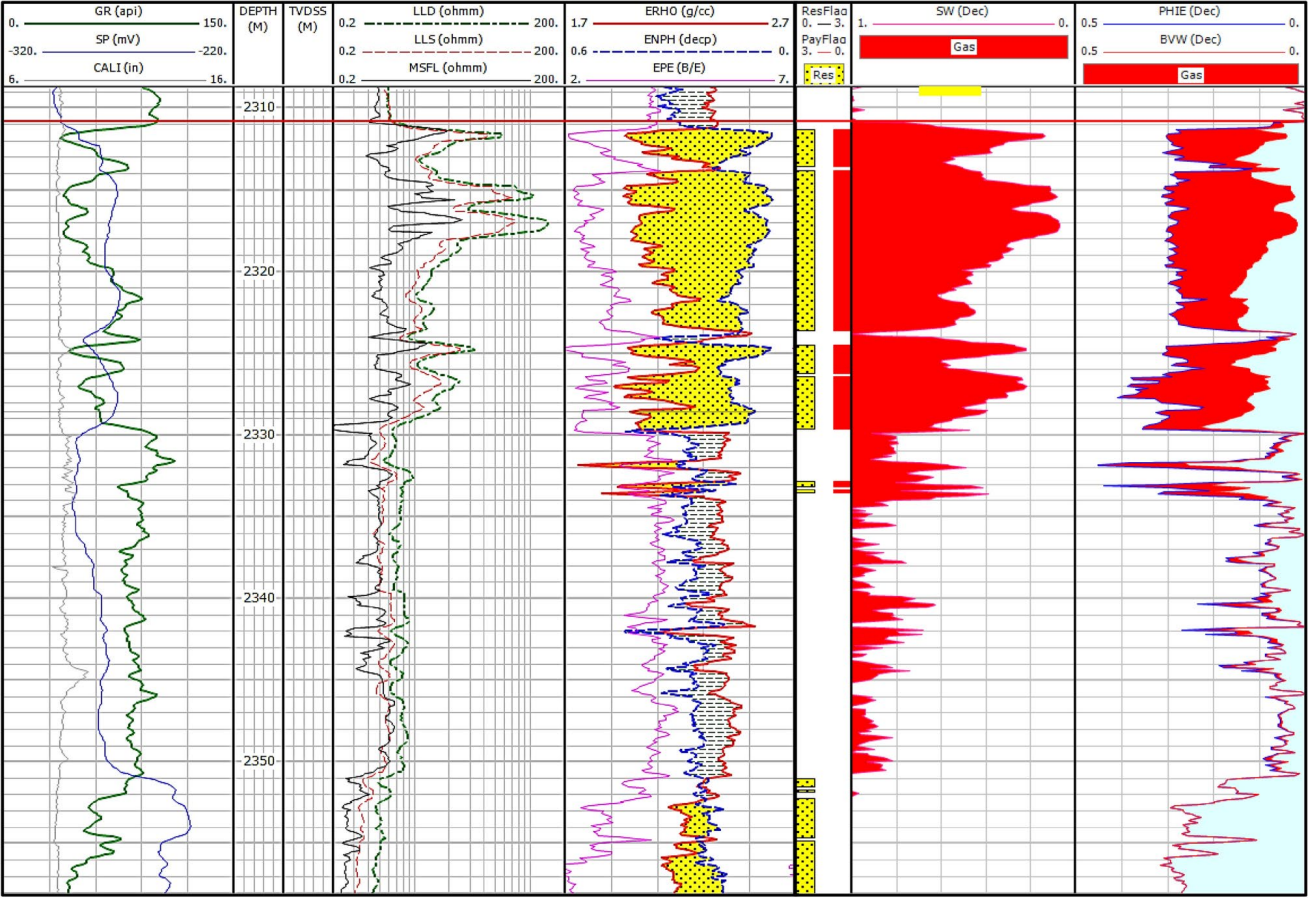
Comprehensive petrophysical analysis of Abu Madi Formation in the SAEN-2 well.

The Clay types were determined using the spectral gamma-ray, SGR Log, especially the Th-K cross-plot^48,49^, revealing the prevalence of mica in the SAEN-2 well (Fig. 10a), while mixed layer and illite clay minerals dominate in the SAEN-9 well (Fig. 10b). Therefore, it is indicated that the clay content changes laterally due to the change of the diagenetic history from the SAEN-2 well to the SAEN-9 well to the northeast of the studied area. This underscores the significance of clay type in changing the reservoir quality from the SAEN-2 well to the SAEN-9 well with better reservoir quality for the SEAN-2 well as indicated from the core data and the triple combo plots (Figs. 11, 12).

#### Petrophysical evaluation

**Fig. 13:**
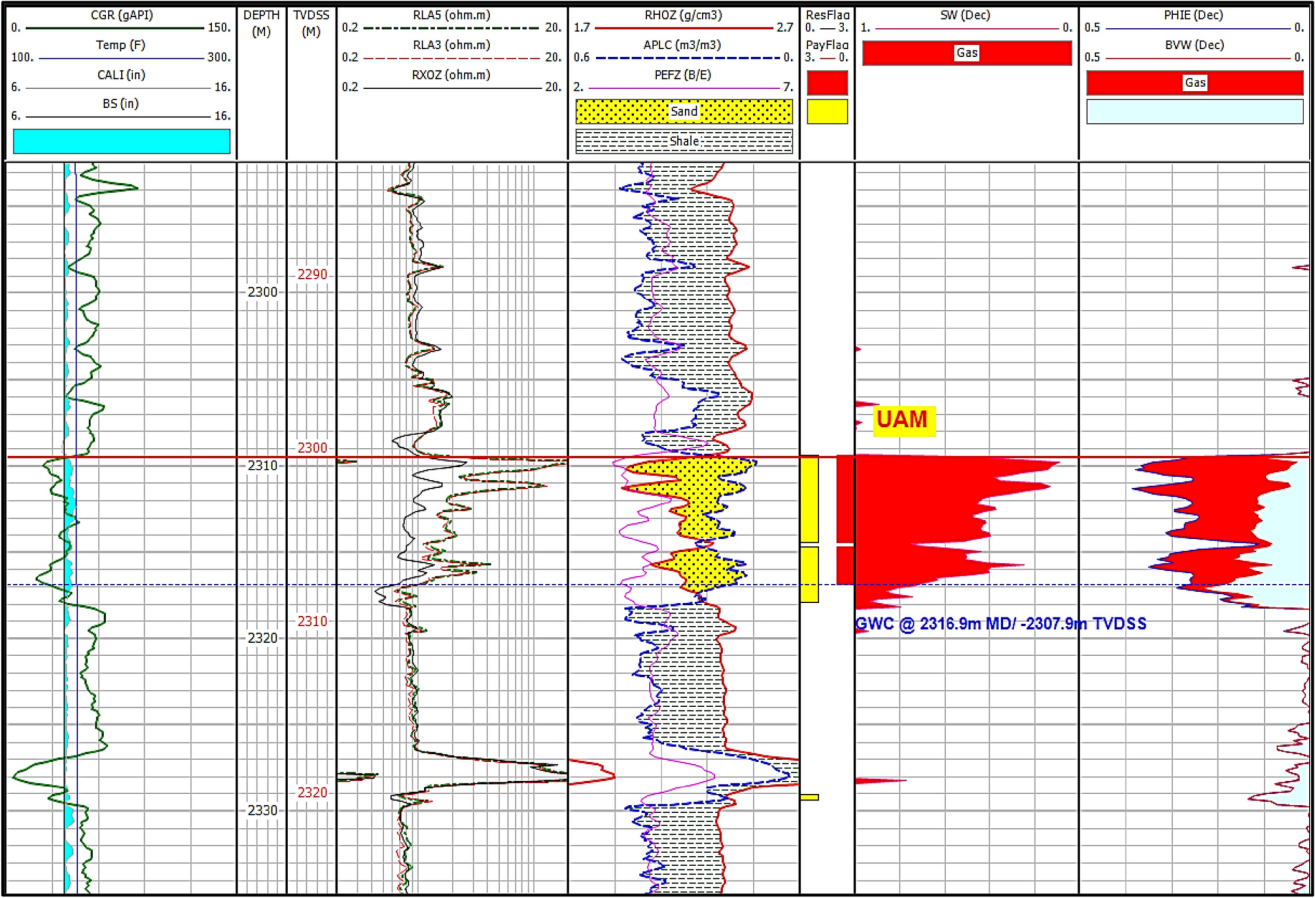
Comprehensive petrophysical analysis of Abu Madi Formation in the SAEN-9 well.

Gamma-ray logs distinguish non-pay shaly intervals from promising sandstones based on the high gamma-ray values which indicate shale, in the form of laminar and/or dispersed pattern, while the low GR values suggesting sandstone intervals^56–60^. In addition, Neutron, density, and photoelectric logs help in the lithology identification, while the crossover between the density and neutron logs assesses the sandstone reservoir quality, with higher crossover indicating gas-bearing sandstone intervals, while the separation between the Rxo and Rt signaling the presence of hydrocarbons. Pickett plot has been utilized, considering the porosity and the true resistivity, showing that the Rw = 0.22 ohmm (Fig. 11). Figures 12, 13 illustrates the petrophysical analysis of the Abu Madi Formation, highlighting the high-quality sandstone in yellow-shaded intervals within the Abu Madi reservoir. The reservoir quality varies between the SAEN-2 and SAEN-9 wells, where the former one has the highest reservoir potentiality in the field, due to good reservoir thickness compared to the latter one which has the lowest quality. For the present study, the petrophysical evaluation was performed considering cutoff parameters (Sw < 70%, V_sh_ < 35%, and ∅ > 6%). Table 4 represents the main petrophysical properties of the Abu Madi Formation in the studied wells, with raw and conditioned logging curves depicted in tracks 1, 4, and 5 (Fig. 12).

**Table 4:** The obtained reservoir parameters of the Abu Madi Formation in the studied wells.

Well	Net pay (m)	Av Phi (%)	Av V_sh_ (%)	Av Sw (%)
SAEN-2	16.6	24.9	25.1	31.7
SAEN-4	10.0	23.3	23.2	33.8
SAEN-6	7.7	30.4	28.4	64.0
SAEN-9	7.3	24.0	24.7	42.0

**Rw = 0.22 ohmm at 28 kppm NaCl concentration and 75°F.

Results of the analysis are presented in tracks 6, 7, and 8, delineating the reservoir and net pay, water and gas saturation, low shale content, and effective porosity. This high reservoir quality with minimal compaction is in accordance with that published by many authors concerning the carbonate and shaly sandstone reservoirs^61–65,66–70^.

**Fig. 14:**
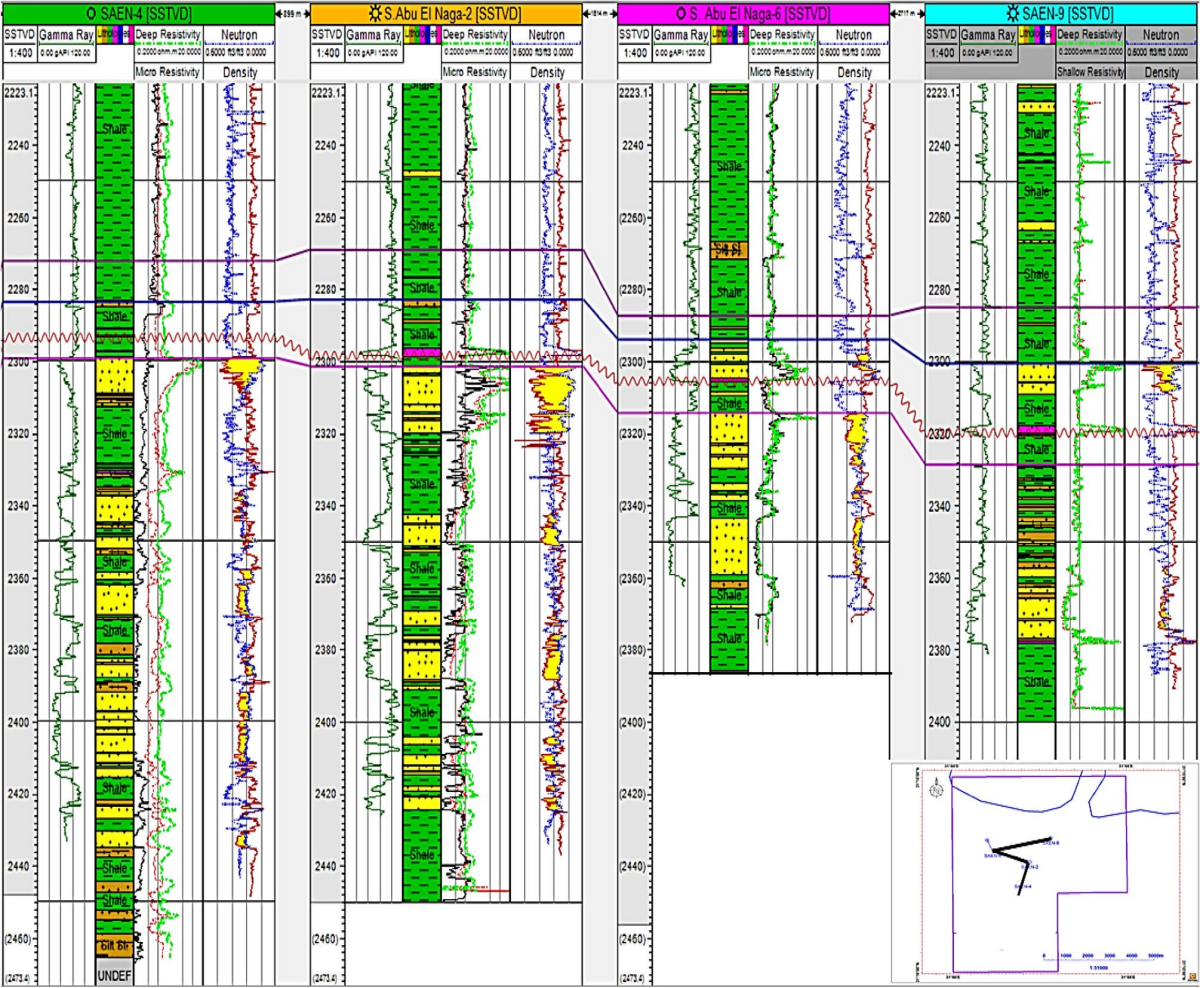
A correlation between the triple combo of the various SAEN wells indicated the most promising reservoir potentiality in SAEN-2 well, and the least potentiality to the northeast in SAEN-9 well.

Note: GR (gamma-ray), SP (spontaneous potential), CAL (caliper), MSFL, LLS, and LLD (are the micro spherical, shallow, and deep resistivities), ERHO (density), ENPH (neutron), EPE (photoelectric factor), Sw (water saturation), PHIE (effective porosity), and BVW (bulk volume of water).

Note: CGR (computed gamma-ray), Temp (Temperature), CAL (caliper), BS (bit size), RXOZ, RLA3, and RLA5 (micro spherical, shallow, and deep resistivities), RHOZ (density), APLC (neutron), PEFZ (photoelectric factor), Sw (water saturation), PHIE (effective porosity), and BVW (bulk volume of water).

In the SAEN-2 well, the Abu Madi reservoir shows an average effective porosity of 24.9% and a water saturation of 31.7%, while the SAEN-9 well displays the effective porosity averaging 24.0% and water saturation averaging 42.0% (Fig. 13). Also, the shale volume (V_sh_) varies between 23.2% in SAEN-4 well and 28.4% in SAEN-6 well, i.e., the shale volume for the Abu Madi reservoir in the various wells is less than the shale volume cutoff value (Table 4). In Fig. 14, the litho-saturation (triple combo) plots of the various SAEN wells have been correlated to compare between the gamma-ray, density-neutron, and resistivity logs to define the highest potentiality in the SAEN Field. It is indicated that, further exploration is not recommended to the northeast direction, and that the best reservoir quality is situated in the center of the field as revealed from Table 4.

## Conclusions

Examining the findings of this study on the Messinian Abu Madi reservoir within the South Abu El Naga Gas (SAEN) Field, Nile Delta, reveals that a multi-faceted approach integrating well log analysis, core data, and petrophysical evaluations has yielded valuable insights into the reservoir properties of the Abu Madi clastics. The well log analysis revealed that the Abu Madi Formation primarily comprises sandstones with dispersed clays and laminated shales.

1. Notably, variations between the petrophysical parameters of the SAEN-2 (net pay = 16.6 m, av. Phi = 24.9%, av. V_sh_ = 25.1%, and av. Sw = 31.7%) and SAEN-9 (net pay = 7.3 m, av. Phi = 24.0%, av. V_sh_ = 24.7%, and av. Sw = 42.0%) wells were observed, with the SAEN-2 well exhibiting thicker pay zones compared to the SAEN-9 well.
2. Petrography confirmed the dominance of quartz arenite sandstones with minimal clay and carbonate content, showcasing porosity levels ranging from 20 to 30%. However, we noted significant amounts of clay content, particularly in the SAEN-9 well, which contained higher water-saturated sandstones and thinner pay zones. Further analysis of core data revealed four distinct reservoir rock types (RRTs) within the reservoir.
3. RRT4 is assigned as the most promising rock type (av. ∅_He_ = 0.26, av. k_H_ = 3678 mD, av. RQI = 3.670 μm, av. FZI = 10.2 μm, av. R_35_ = 39.42 μm, and av. Sw = 50.68%); it is characterized by very good Flow Zone Indicator (FZI) values, indicating the highest sandstone reservoir quality with maximum porosity, low clay content, and minimal compaction.
4. On the other side, RRT1 had the worst reservoir quality (av. ∅_He_ = 0.20, av. k_H_ = 142.3 mD, av. RQI = 0.629 μm, av. FZI = 2.245 μm, av. R_35_ = 5.55 μm, and av. Sw = 61.42%), with few holes, a lot of clay, and a lot of compaction. Integrating all this data allowed the identification of four distinct hydraulic flow units for the Abu Madi Formation.
5. Focusing exploration and development efforts on zones with favorable flow characteristics, such as high FZI and low clay content, holds the potential to maximize production from this reservoir.

## Data Availability

Data will be available on reasonable request based on permission from the Egyptian General Petroleum Corporation by contacting the corresponding author: Bassem S Nabawy; bsnabawy@yahoo.co.uk; bs.nabawy@nrc.sci.eg

## Abbreviations

APLC: Neutron density
BS: Bit size
BVW: Bulk volume of water
CAL: Caliper
CGR: Computed gamma-ray
FZI: Flow zone indicator
GR: Gamma-ray
HFU: Hydraulic flow unit
PEF: Photoelectric Factor
PHIE: Effective porosity
RHOZ: Bulk density from well log
RLA3: Medium resistivity
RLA5: Deep resistivities
RXOZ: Shallow resistivity
SAEN: South Abu El Naga
SEM: Scanning electron microscopy
SGR: Spectral gamma-ray
Sw: Water saturation
XRD: X-ray diffraction

## List of symbols

Φe: Effective porosity
Ø_N_: Neutron porosity
Ø_D_: Density porosity
Ø_Nsh_: Neutron porosity of shale
Ø_Dsh_: Density porosity of shale
Ø_T_: Total porosity
k_H_: Horizontal permeability
k_V_: Vertical permeability
RQI: Reservoir quality index
R_35_: Effective pore radius
RRT: Reservoir rock type
ρ_g_: Grain density
Vsh: Shale volume
